# Multifaceted Immunomodulatory Effects of the BTK Inhibitors Ibrutinib and Acalabrutinib on Different Immune Cell Subsets – Beyond B Lymphocytes

**DOI:** 10.3389/fcell.2021.727531

**Published:** 2021-08-13

**Authors:** Sining Zhu, Samantha Gokhale, Jaeyong Jung, Eris Spirollari, Jemmie Tsai, Johann Arceo, Ben Wang Wu, Eton Victor, Ping Xie

**Affiliations:** ^1^Department of Cell Biology and Neuroscience, Rutgers University, Piscataway, NJ, United States; ^2^Graduate Program in Cellular and Molecular Pharmacology, Rutgers University, Piscataway, NJ, United States; ^3^Rutgers Cancer Institute of New Jersey, New Brunswick, NJ, United States

**Keywords:** BTK, ibrutinib, acalabrutinib, immune cell subsets, immune responses, inflammation, cancers, COVID-19

## Abstract

The clinical success of the two BTK inhibitors, ibrutinib and acalabrutinib, represents a major breakthrough in the treatment of chronic lymphocytic leukemia (CLL) and has also revolutionized the treatment options for other B cell malignancies. Increasing evidence indicates that in addition to their direct effects on B lymphocytes, both BTK inhibitors also directly impact the homeostasis, phenotype and function of many other cell subsets of the immune system, which contribute to their high efficacy as well as adverse effects observed in CLL patients. In this review, we attempt to provide an overview on the overlapping and differential effects of ibrutinib and acalabrutinib on specific receptor signaling pathways in different immune cell subsets other than B cells, including T cells, NK cells, monocytes, macrophages, granulocytes, myeloid-derived suppressor cells, dendritic cells, osteoclasts, mast cells and platelets. The shared and distinct effects of ibrutinib *versus* acalabrutinib are mediated through BTK-dependent and BTK-independent mechanisms, respectively. Such immunomodulatory effects of the two drugs have fueled myriad explorations of their repurposing opportunities for the treatment of a wide variety of other human diseases involving immune dysregulation.

## Introduction

The Bruton’s tyrosine kinase (BTK) inhibitors, along with other targeted drugs such as the BCL-2 inhibitors, have fundamentally changed the treatment landscape of chronic lymphocytic leukemia (CLL) and have been transforming the treatment algorithms of other B cell malignancies (Pal Singh et al., 2018; Lucas and Woyach, 2019; Rhodes and Barrientos, 2020). BTK, a member of the TEC kinase family, was initially identified as a non-receptor protein tyrosine kinase that is inactive in patients with the inherited immunodeficiency disease X-linked agammaglobulinemia (XLA) (Hendriks et al., 2014; Pal Singh et al., 2018; Rip et al., 2018). It is required for B cell receptor (BCR) signaling, and therefore plays essential roles in B cell development, survival, proliferation, differentiation and activation (Pal Singh et al., 2018; Rip et al., 2018; Wen et al., 2021). Upon BCR activation, BTK forms a signaling complex together with SYK, VAV, PI3K, SLP65 and PLCγ2. BTK phosphorylates PLCγ2 to activate the transcription factors nuclear factor-κB (NF-κB), nuclear factor of activated T cells (NF-AT), as well as ERK1 and ERK2, which in turn mediate downstream functional responses (Hendriks et al., 2014; Pal Singh et al., 2018; Rip et al., 2018; Wen et al., 2021). In addition to BCR, BTK also regulates the signaling pathways of chemokine receptors in B cells, including CXCR4 and CXCR5, which play pivotal roles in B cell chemotaxis and migration (de Gorter et al., 2007; Hendriks et al., 2014). Overexpression and constitutive activation of BTK have been reported in multiple types of B cell malignancies (Herman et al., 2011; Hendriks et al., 2014; Merolle et al., 2018). Overall, BTK is crucial in the pathogenesis of B cell malignancies and is critically involved in malignant B cell survival, proliferation and migration, and thus has been recognized as a prime therapeutic target for B cell malignancies (Hendriks et al., 2014; Pal Singh et al., 2018).

Ibrutinib is the first-in-class oral inhibitor of BTK. Ibrutinib irreversibly inactivates BTK by covalently binding to Cys481 in the ATP-binding site of BTK (Hendriks et al., 2014; Pal Singh et al., 2018). Ibrutinib has shown potent anti-tumor activity in both indolent and aggressive B cell lymphomas and achieved unprecedently high response rates in patients with CLL (>90% response rate) and mantle cell lymphoma (MCL; >80% response rate) in clinical trials. Targeting BTK in CLL and MCL with ibrutinib results in direct inhibition of cell proliferation and homing/migration due to disruption of BCR and chemokine receptor signaling (Herman et al., 2011; de Rooij et al., 2012; Ponader et al., 2012; Hendriks et al., 2014; Pal Singh et al., 2018). Based on the clinical evidence, ibrutinib has been given the United States Food and Drug Administration (FDA) approval for the treatment of multiple B cell malignances, including CLL/small lymphocytic lymphoma (SLL), MCL, marginal zone lymphoma (MZL) and Waldenstrom macroglobulinemia (WM) (Hendriks et al., 2014; Pal Singh et al., 2018; Zi et al., 2019; Bond and Maddocks, 2020; Castillo et al., 2020b; Grimont et al., 2020; Hanna et al., 2020; Noy et al., 2020; Treon et al., 2021). Furthermore, ibrutinib, as monotherapy or in combination therapies with other targeted drugs (such as anti-CD20, anti-PD-1/PD-L1 and inhibitors of Bcl-2, PI-3Kδ or proteosome), has demonstrated efficacy in patients with diffuse large B-cell lymphoma (DLBCL), follicular lymphoma (FL), Richter’s transformation (RT), multiple myeloma (MM), B cell pro-lymphocytic leukemia (B-PLL), acute lymphoblastic leukemia (ALL), lymphoproliferative disorders (LPD), and primary and secondary central nervous system lymphomas (PCNSL/SCNSL) in recent clinical trials (Hendriks et al., 2014; Pal Singh et al., 2018; Chari et al., 2020; Chen et al., 2020; Fowler et al., 2020; Oka et al., 2020; Schaffer et al., 2020; Graf et al., 2021; Hodkinson et al., 2021; Lewis et al., 2021). However, a unique set of toxicities has also been reported, even though ibrutinib is generally more tolerable than chemoimmunotherapy (CIT) regimens. Common adverse effects of ibrutinib include bleeding, atrial fibrillation, hypertension, neutropenia, arthralgias, myalgias, headache, diarrhea, nausea, fatigue, rash and infection (Bitar et al., 2018; Ball et al., 2020; Kin and Schiffer, 2020; Lasica and Tam, 2020; Lipsky and Lamanna, 2020; Los-Arcos et al., 2020; Pileri et al., 2020; Rhodes et al., 2020; Estupinan et al., 2021; Pellegrini et al., 2021; Steingrimsson et al., 2021). These side effects are mediated by both on-target inhibition of BTK and variable off-target inhibition of other kinases such as interleukin-2-inducible T-cell kinase (ITK), tyrosine kinase expressed in hepatocellular carcinoma (TEC), CSK, SRC, BMX, JAK3, epidermal growth factor receptor (EGFR), c-Kit and platelet-derived growth factor receptor (PDGFR), etc. (Bitar et al., 2018; Liclican et al., 2020; Lipsky and Lamanna, 2020; Sibaud et al., 2020; Estupinan et al., 2021). For example, the effect of ibrutinib on atrial fibrillation is caused by its off-target inhibition of CSK (Xiao et al., 2020), while the skin toxicities of ibrutinib likely involve its inhibition of EGFR as the symptoms overlap with those caused by selective EGFR inhibitors (Singer et al., 2019). The remarkable clinical efficacy of ibrutinib has thus generated great interests to develop the next generation of BTK inhibitors to improve target specificity and reduce off-target toxicities.

Acalabrutinib (ACP-196), a representative second-generation BTK inhibitor, is highly specific for BTK and has minimal effects on other kinases (Byrd et al., 2016; Wu et al., 2016; Barf et al., 2017; Danilov and Persky, 2020). Similar to ibrutinib, acalabrutinib also irreversibly binds to Cys481 located in the ATP-binding site of BTK (Wu et al., 2016; Barf et al., 2017). Compared to ibrutinib, acalabrutinib is more potent, demonstrates higher biochemical and cellular selectivity, and has a faster oral absorption and a shorter half-life (Covey et al., 2015; Byrd et al., 2016; Wu et al., 2016; Barf et al., 2017; Davids et al., 2020; Wen et al., 2021). Due to its high efficacy and improved safety profile, acalabrutinib was granted accelerated approval by FDA in 2017 for the treatment of adult patients with MCL who have received at least one prior therapy (Wang et al., 2018; Telford et al., 2019; Witzig and Inwards, 2019; Bond and Maddocks, 2020; Danilov and Persky, 2020; Morabito et al., 2020). In November 2019, it was also approved for the treatment of adult patients with CLL/SLL (Danilov and Persky, 2020; Davids et al., 2020; Ghia et al., 2020, 2021; Isaac and Mato, 2020; Sharman et al., 2020; Fakhri and Andreadis, 2021). Importantly, a multinational phase I/II study demonstrated the efficacy of acalabrutinib in ibrutinib-intolerant CLL patients, verifying its reduced toxicities compared to ibrutinib (Isaac and Mato, 2020). Clinical trials have also shown the efficacy of acalabrutinib monotherapy in patients with WM (Castillo et al., 2020a; Castillo and Treon, 2020; Owen et al., 2020), which bodes well for additional FDA approval of acalabrutinib in the treatment of WM. A number of ongoing clinical trials are evaluating the effects of acalabrutinib, as monotherapy or in combination therapies with other regimens, in patients with DLBCL (monotherapy or in combination with R-CHOP, KRT-232 or vistusertib), FL (in combination with rituximab or pembrolizumab), MZL (in combination with tafasitamab), MM (monotherapy or in combination with dexamethasone), B-ALL (in combination with ACP-319), post-transplant LPD (in combination with rituximab), PCNSL and SCNSL (in combination with durvalumab)^1^. Despite its improved specificity and toxicity profile, common adverse effects of acalabrutinib that have been documented include headache, diarrhea, fatigue, myalgias, cough, neutropenia, nausea, skin rash and infection (Awan et al., 2019; Khan and O’Brien, 2019; Byrd et al., 2020; Owen et al., 2020; Sibaud et al., 2020; Delgado et al., 2021), prompting better understanding of the underlying mechanisms.

Since their approval by FDA, ibrutinib and acalabrutinib have significantly altered the clinical course and substantially prolonged the progression-free survival of CLL and MCL patients, especially in high-risk patients (Bond et al., 2019; Liu and Zhao, 2019; Lucas and Woyach, 2019; Telford et al., 2019; Rhodes and Barrientos, 2020). In 2019, both drugs were upgraded from being a “great treatment option” to the “preferred choice” for all lines of treatment in CLL and relapsed MCL after multiple randomized clinical trials proved their superiority compared to conventional CIT regimens, leading to a paradigm shift to chemotherapy-free treatment (Liu and Zhao, 2019; Telford et al., 2019; Bond and Maddocks, 2020; Hanna et al., 2020; Iovino and Shadman, 2020; Rhodes and Barrientos, 2020). It is increasingly clear that in addition to their direct effects on B cells, both BTK inhibitors also directly impact the phenotype and function of many other cell subsets of the immune system, which contribute to their high efficacy as well as adverse effects observed in CLL and MCL patients. Interestingly, such immunomodulatory effects of ibrutinib and acalabrutinib are being exploited to treat a variety of other human diseases, including other hematological malignancies, solid tumors, graft-*versus*-host disease (GVHD), autoimmune disorders, atherothrombosis, allergy and infectious diseases (Zaitseva et al., 2014; Pillinger et al., 2015; Rushworth et al., 2015; Miklos et al., 2017; Molina-Cerrillo et al., 2017; Dispenza et al., 2018; Florence et al., 2018; Mamand et al., 2018; Rip et al., 2018; Allchin et al., 2019; de Porto et al., 2019; Goldmann et al., 2019; O’Riordan et al., 2019; Riccio et al., 2019; Varikuti et al., 2019; Waller et al., 2019; Hu et al., 2020; Lorenzo-Vizcaya et al., 2020; Metzler et al., 2020; Purvis et al., 2020; Shaker et al., 2020; Teusink-Cross et al., 2020; Nadeem et al., 2021; Nicolson et al., 2021; Wen et al., 2021). Most notably, the repositioning therapeutic potential of BTK inhibitors has been demonstrated by recent application of acalabrutinib in the management of severe respiratory syndrome in patients with COVID-19 (Rada et al., 2020; Roschewski et al., 2020; Treon et al., 2020; Benner and Carson, 2021; Fiorcari et al., 2021; McGee et al., 2021).

Numerous studies have analyzed and reviewed the effects of ibrutinib and acalabrutinib on receptor signaling pathways in normal and malignant B cells. However, so far direct comparison of the effects of these two BTK inhibitors on signaling pathways in non-B immune cell subsets is very limited in the published literature. In this review, we compare the effects of ibrutinib and acalabrutinib on receptor signaling pathways in different immune cell subsets beyond B lymphocytes. Such understanding will provide a rationale to develop optimal combination therapies to achieve much deeper and longer remission in B cell malignancies and will inform future efforts on managing immune-mediated adverse effects and expanding the clinical applications of both drugs to the treatment of other human diseases.

## General Immunomodulatory Effects of Ibrutinib and Acalabrutinib

Mounting evidence obtained from ibrutinib-treated CLL and MCL patients has revealed that in addition to depleting malignant B cells, ibrutinib has compound immunomodulatory effects on the cytokine/chemokine network and a variety of immune cell subsets of both the adaptive and innate immune systems, including CD4 and CD8 T cells, natural killer (NK) cells, monocytes, macrophages, granulocytes, myeloid-derived suppressor cells (MDSCs), dendritic cells (DCs), osteoclasts, mast cells and platelets (Berglof et al., 2015; Long et al., 2017; Pleyer et al., 2018; Mhibik et al., 2019; Cadot et al., 2020; Maffei et al., 2020; Solman et al., 2020, 2021). For example, ibrutinib treatment generally decreases the abnormally high counts of chronically activated, exhausted and effector memory T cells as well as immunosuppressive regulatory T cells (Treg) and myeloid-derived suppressor cells (MDSCs), while restoring the low counts of innate cell subsets such as circulating monocytes and plasmacytoid DCs in CLL patients toward the healthy donor range, thereby rescues both T cell and myeloid cell defects associated with CLL (Pleyer et al., 2018; Parry et al., 2019; Solman et al., 2020, 2021). Interestingly, the effects of ibrutinib on T cells are mainly mediated by its off-target inhibition on ITK, a critical signaling component in T cells and NK cells (Long et al., 2017; Flinsenberg et al., 2019; Mhibik et al., 2019). Given the high specificity of acalabrutinib on BTK and its minimal off-target inhibition of other kinases, the immunomodulatory profile of acalabrutinib is predicted to be distinct from that observed for ibrutinib (Patel et al., 2017). One clinically important difference is that ibrutinib affects antibody-dependent cellular cytotoxicity (ADCC) in NK cells but acalabrutinib does not, leading to much higher potential for acalabrutinib than ibrutinib in combination therapies with anti-CD20 (such as rituximab and obinutuzumab) and many other immunotherapeutic antibodies (Kohrt et al., 2014; Hassenrück et al., 2018; Woyach et al., 2020). However, BTK is not only expressed in B cells but also expressed in other immune cell subsets, including monocytes, macrophages, granulocytes, DCs, MDSCs, osteoclasts, mast cells, megakaryocytes, platelets, NK cells and T cells (Guendel et al., 2015; Weber et al., 2017; Pal Singh et al., 2018; Rip et al., 2018; Xia et al., 2019). Therefore, both BTK inhibitors may affect the homeostasis or function of these immune cell subsets via BTK-dependent mechanisms as well. Here we attempt to provide an overview of current understanding of the overlapping and differential effects of ibrutinib and acalabrutinib on different signaling pathways in various immune cell subsets other than B lymphocytes.

## T Cells and Chimeric Antigen Receptor-T (CAR-T) Cells

With acalabrutinib, circulating CD4 T cell counts are not changed, while CD8 T cell counts are decreased after 15 cycles of treatment in CLL patients (Byrd et al., 2016). In contrast, ibrutinib treatment exhibits profound effects on the T cell compartments in both CLL and MCL patients, which vary depending on the length of treatment and the time-point examined (Maharaj et al., 2017; Man and Henley, 2019; Cadot et al., 2020). CLL patients usually have immunocompromised T cell compartments with elevated frequencies of chronically activated, exhausted and effector memory T cells and immunosuppressive Tregs. Ibrutinib monotherapy generally improves T cell compartments in CLL patients (Cubillos-Zapata et al., 2016; Yin et al., 2017; Mhibik et al., 2019; Parry et al., 2019; Cadot et al., 2020; Solman et al., 2020, 2021). After 4 weeks of ibrutinib treatment, T cell numbers and the percentage of CD4 T cells, memory CD8 (CD45RO+) T cells and Tregs as well as IL-10 concentration are reduced in CLL patients (Podhorecka et al., 2017). After 8 weeks of treatment, the numbers of naïve and effector/memory CD8 and CD4 T cells are markedly increased, while the expression of immunoinhibitory PD-1 and CTLA-4 is significantly reduced on T cells in CLL patients (Long et al., 2017; Cadot et al., 2020). After 12 and 24 weeks of treatment, decreased frequency of T_H_17 cells and overall T cell numbers as well as reduced expression of PD-1 and activation markers on T cells are observed in CLL patients (Cubillos-Zapata et al., 2016; Niemann et al., 2016). After the first year of ibrutinib treatment, the pathologically high frequencies of exhausted or chronically activated effector/memory CD4 and CD8 T cells as well as Tregs are reduced, while naive T cells are preserved and T cell receptor (TCR) repertoire diversity is significantly increased in CLL patients (Ryan et al., 2016; Yin et al., 2017; Mhibik et al., 2019; Solman et al., 2020). Interestingly, a reduction in GATA3-expressing T_H_2 cells but no change in T-bet-expressing T_H_1 cells have been noticed in the patients (Ryan et al., 2016). Moreover, ibrutinib downregulates the expression of inhibitory receptors and restores the functions of patient-derived T cells, including proliferation, degranulation, and cytokine secretion (Ryan et al., 2016; Mhibik et al., 2019; Solman et al., 2020). In CLL patients receiving ibrutinib therapy for 2 to 4 years, naïve T cells remain within healthy donor range, PD-1 expression is consistently reduced on chronically activated CD8 T cells, and production of IFNγ and TNFα by antigen-specific CD8 T cells is enhanced following stimulation with CMV or EBV peptides, suggesting that long-term treatment of ibrutinib may reverse the exhausted T cell phenotype (Parry et al., 2019; Solman et al., 2021). In MCL patients receiving long-term (>12 months) combination therapy with ibrutinib and venetoclax, increased frequencies of CD4 and CD8 effector and central memory T cells as well as normalized T cell cytokine production have been documented, suggesting the recovery of T cell compartments (Davis et al., 2020). Overall, ibrutinib exhibits beneficial immunomodulatory effects on T cell compartments and promotes the reconstitution of adaptive immunity in both CLL and MCL patients.

Mechanistic investigation has revealed that the majority of ibrutinib-induced modulatory effects on T cell compartments are mediated through its inhibition of ITK in TCR signaling pathways, including reduced T_H_2 polarization, altered T_H_17 and Treg balance, and activation-induced cytokine production as well as cell death (Table 1). ITK is highly expressed in T cells and regulates TCR-induced proliferation, activation and cytokine production (Berglof et al., 2015). TCR signaling is primarily dependent on ITK in T_H_2 cells, but only partially dependent on ITK in T_H_1 and CD8 T cells due to the presence of another redundant kinase RLK (Dubovsky et al., 2013; Berglof et al., 2015; Mhibik et al., 2019). Consistent with this notion, a shift toward the T_H_1 phenotype, reduced T_H_2 cell numbers and decreased production of T_H_2 cytokines have been detected in ibrutinib-treated CLL patients (Dubovsky et al., 2013; Ryan et al., 2016; Mhibik et al., 2019; Solman et al., 2020). Interestingly, ibrutinib treatment also promotes an anti-tumor T_H_1 phenotype of Vγ9Vδ2 T cells via an ITK-dependent mechanism and rescues the dysfunction of autologous Vγ9Vδ2 T cells in CLL patients, resulting in potent cytotoxicity toward malignant B cells (de Weerdt et al., 2018). Ibrutinib irreversibly binds to ITK in conventional T cells and Vγ9Vδ2 T cells obtained from the patients (Dubovsky et al., 2013; de Weerdt et al., 2018). ITK also critically regulates the T_H_17 *versus* Treg differentiation (Mhibik et al., 2019). *Itk*^–/–^ CD4 T cells preferentially differentiate into Tregs both *in vivo* and under conditions favoring T_H_17 differentiation *in vitro*, while T cells from a patient with *ITK* mutation exhibit decreased production of T_H_17-associated cytokines IL-17A, IL-22 and GM-CSF (Gomez-Rodriguez et al., 2014; Eken et al., 2019; Mhibik et al., 2019). Recapitulating the effects of *ITK* deficiency, ibrutinib suppresses human CD4 T cells of healthy donors from differentiating into T_H_17 cells *in vitro* and reduces the *in vivo* frequencies of both T_H_17 and Tregs as well as the serum levels of T_H_17-associated cytokines IL-17A, IL-21 and IL-23 in CLL patients (Niemann et al., 2016; Eken et al., 2019; Mhibik et al., 2019). Furthermore, T cells from *Itk*^–/–^ mice exhibit diminished activation-induced cell death (AICD) with defective FAS ligand (FASL) expression (Miller and Berg, 2002; Sun et al., 2015). Mirroring this phenotype, ibrutinib but not acalabrutinib inhibits AICD in human T cells by reducing the upregulation of FASL, and increases CD4 and CD8 T cell numbers especially the effector/effector memory subsets in CLL patients (Long et al., 2017). Thus, ibrutinib-mediated inhibition of ITK-dependent signaling pathways in T cells have been elucidated by examining *ITK*-deficient model systems and by comparing the effects of ibrutinib and acalabrutinib on T cells.

**TABLE 1:** Effects of ibrutinib and acalabrutinib on T cells and NK cells.

Cells	Inhibitor	Target	Signaling pathway	Effects	References
T cells	Acalabrutinib	BTK	TCR-BTK-PLCγ1	Decreases CD8 T cell counts after 15 cycles of treatment in CLL patients	Byrd et al., 2016
				Suppresses T cell proliferation after stimulation with CD3 and CD28	Xia et al., 2019
				Reduces the expansion of WT donor T cells and ameliorates bone marrow destruction and aplastic anemia in recipient mice	Xia et al., 2019
				Downregulates PD-1 and CTLA-4 expression on T cells in CLL patients	Long et al., 2017
	Ibrutinib	BTK	TCR-BTK-PLCγ1	Downregulates PD-1 and CTLA-4 expression on T cells in CLL patients	Cubillos-Zapata et al., 2016; [Bibr B162][Bibr B152]; Long et al., 2017
			TLR7-BTK-STAT3	Inhibits imiquimod-induced IL-17 production in dermal γδ T cells	Nadeem et al., 2020
		ITK	TCR-ITK-PLCγ1-NF-κB/MAPK/NFAT/STAT6	Preserves naïve T cells, increasing diversification of the TCR repertoire and decreasing exhausted T cells and Tregs in CLL patients	Parry et al., 2019; [Bibr B200][Bibr B39]; [Bibr B138][Bibr B230]; Cadot et al., 2020
				Increases CD4 and CD8 T cell numbers and reduces Treg/CD4 T cell ratio in CLL patients	Long et al., 2017
				Enhances Th1 response and impairs Th2 polarization	Dubovsky et al., 2013; Solman et al., 2020
					Ryan et al., 2016; Mhibik et al., 2019
				Reduces Th17 differentiation and frequency	Niemann et al., 2016; Podhorecka et al., 2017
				Reduces Treg cell frequency and reduces serum level of IL-10	Podhorecka et al., 2017
					
				Inhibits FASL expression and AICD	Long et al., 2017
				Enhances production of IFNγ and TNFα by CD8 T cells after stimulation with CMV/EBV peptides	Parry et al., 2019
				Reduces the frequency of memory CD8 T cells	Podhorecka et al., 2017
				Promotes an anti-tumor Th1 phenotype of Vγ9Vδ2 T cells	de Weerdt et al., 2018
				Inhibits γδ T cell activation and CD107a degranulation induced by phosphoantigens or anti-CD3	Risnik et al., 2020
CAR-T cells	Acalabrutinib	BTK?	Indirectly mediated by downregulation of PD-1 and CTLA-4?	Improves CAR-T cell effector function in prolonged stimulation assays	Qin et al., 2020
				Improves CAR-T cell-mediated clearance of CD19+ tumor in mouse xenograft models	Qin et al., 2020
	Ibrutinib	BTK/ITK?	Indirectly mediated by downregulation of PD-1 and CTLA-4?	Improves the *ex vivo* and *in vivo* expansion of CAR-T cells derived from ibrutinib-treated CLL patients	Fraietta et al., 2016
				Enhances the anti-tumor efficacy of CAR-T cells and reduces cytokine release syndrome (CRS) when given concurrently with CAR-T cells in R/R CLL patients	Gauthier et al., 2020
				Enhances the killing activities of anti-CD19 CAR-T cells *in vitro* and in mouse xenograft models	Fraietta et al., 2016; Ruella et al., 2016
				Improves CAR-T cell engraftment, tumor clearance and long-term remission in mouse xenograft models of CLL, ALL and MCL	Fraietta et al., 2016; Ruella et al., 2016
				Inhibits the production of inflammatory cytokines from CAR-T cells in a MCL xenograft model	Ruella et al., 2017
				Increases cell viability and expansion of CLL patient-derived CAR-T cells after *ex vivo* treatment	Fan et al., 2021
				Decreases the expression of PD-1, TIM-3 and LAG-3 and enriches CAR-T cells with less-differentiated naïve-like phenotype	Fan et al., 2021
				Improves CAR-T cell effector function in prolonged stimulation assays	Qin et al., 2020
		ITK	TCR-ITK-PLCγ1?	Induces gene expression changes of CAR-T cells toward a memory-like, Th1 phenotype	Qin et al., 2020
NK cells	Acalabrutinib		None	Does not affect ADCC	Hassenrück et al., 2018
					Woyach et al., 2020
	Ibrutinib	ITK?	Unclear	Decreases immature CD16- NK cell counts in CLL patients	Solman et al., 2020, 2021
				Inhibits FASL expression and AICD in NK cells	Long et al., 2017
		ITK	FcγRIIIA (CD16)-ITK-PLCγ2	Inhibits ADCC, calcium mobilization, IFNγ production and degranulation in response to opsonized CLL or MCL cells	Bojarczuk et al., 2014; [Bibr B105][Bibr B40]; [Bibr B85][Bibr B68]; Hofland et al., 2019
NKT cells	Ibrutinib	ITK?	Unclear	Reduces the aberrantly elevated NKT cell counts in CLL patients	Solman et al., 2020, 2021

In addition to ITK, BTK is also expressed in T cells and further upregulated in effector and memory T cells (Xia et al., 2019). Recent evidence reveals that BTK is activated and phosphorylated by TCR signaling to promote T cell proliferation and activation by phosphorylating PLCγ1 (Xia et al., 2019). In response to CD3 and CD28 stimulation, *Btk*^–/–^ T cells exhibit defective proliferation and reduced expression of the activation marker CD69 as well as production of cytokines (Xia et al., 2019). Treatment with acalabrutinib (0.1–1 μM) robustly suppresses the proliferation of WT but not *Btk*^–/–^ T cells induced by CD3 and CD28 *in vitro* (Xia et al., 2019). In a mouse model of immune-mediated aplastic anemia, *Btk*^–/–^ donor T cells fail to mount graft-*versus*-host responses and cannot cause bone marrow destruction or blood pancytopenia in recipient mice (Xia et al., 2019). *In vivo* administration of acalabrutinib reduces the expansion of WT donor T cells and ameliorates bone marrow destruction and aplastic anemia in recipient mice (Xia et al., 2019). Interestingly, expression of the immune checkpoints PD-1 and CTLA-4 on T cells in CLL patients is markedly downregulated by both ibrutinib and acalabrutinib (Long et al., 2017), suggesting a common BTK-dependent mechanism. Furthermore, ibrutinib significantly decreases dermal IL-17A-producing γδ T cells in a mouse model of imiquimod-induced psoriatic inflammation by inhibiting the TLR7-BTK-STAT3 signaling pathway (Nadeem et al., 2020), while reducing γδ T cell activation and CD107a degranulation induced by phosphoantigens or anti-CD3 by inhibiting the TCR-ITK signaling pathway (Risnik et al., 2020). Thus, while ibrutinib has unique ITK-dependent mechanisms of action on T cells, both ibrutinib and acalabrutinib can modulate T cell activation and phenotype via BTK-dependent mechanisms (Table 1; Long et al., 2017; Xia et al., 2019; Nadeem et al., 2020). These findings suggest potential applications of both BTK inhibitors for the treatment of human diseases involving T cell abnormalities, including GVHD, autoimmune diseases and cancers.

Both ibrutinib and acalabrutinib also inhibit the phosphorylation of LCK and SRC in human T cells of healthy donors in a dose-dependent manner, but the extent and efficacy of inhibition are very different for the two drugs (Patel et al., 2017). Ibrutinib demonstrates an almost complete inhibition with an EC50 < 0.2 μM and acalabrutinib shows a partial inhibition on the phosphorylation of LCK and SRC with an EC50 > 10 μM (Patel et al., 2017). This suggests that ITK plays a dominant role and BTK may play a supporting role in these signaling events, or alternatively, ibrutinib may have direct off-target inhibition on LCK and SRC, which requires further investigation.

Of clinical importance, emerging evidence indicates that both ibrutinib and acalabrutinib can improve the efficacy of chimeric antigen receptor-T (CAR-T) cells, a promising therapy for B cell malignancies and other human cancers. Impaired T cell fitness and defective T cell compartments of CLL patients often hampers the expansion and function of CAR-T cells (Fraietta et al., 2016; Fan et al., 2021). It is found that pretreatment with ibrutinib (≥1 year and ≥5 cycles) improves the *ex vivo* and *in vivo* expansion of CD19-directed CAR-T cells and decreases the expression of the immunoinhibitory receptor PD-1 on T cells and of the immunoinhibitory ligand CD200 on malignant B cells in CLL patients (Fraietta et al., 2016). Concurrent ibrutinib treatment improves the anti-tumor efficacy of CAR-T cells and reduces cytokine release syndrome (CRS) associated with CAR-T cell therapy in relapsed/refractory (R/R) CLL patients, resulting in improved clinical outcome (Gauthier et al., 2020). In *in vitro* assays and in mouse xenograft models, ibrutinib enhances the killing activities of anti-CD19 CAR-T cells derived from CLL or MCL patients or normal donors, and improves CAR-T cell engraftment, tumor clearance and long-term remission in mice with xenografts of human CLL, ALL or MCL (Fraietta et al., 2016; Ruella et al., 2016). Ibrutinib also inhibits the production of inflammatory cytokines from both CAR-T cells and tumor cells during CAR-T cell therapy in a MCL xenograft model (Ruella et al., 2017). A more recent study reports that *ex vivo* treatment with ibrutinib increases cell viability and expansion of CLL patient-derived CAR-T cells, enriches CAR-T cells with less-differentiated naïve-like phenotype, decreases the expression of the exhaustion markers PD-1, TIM-3 and LAG-3, and enhances the cytokine release capacity of CLL patient-derived CAR-T cells (Fan et al., 2021). Furthermore, *in vitro* treatment with ibrutinib or acalabrutinib improves CAR-T cell effector function in prolonged stimulation assays, while *in vivo* administration of ibrutinib or acalabrutinib improves CAR-T cell-mediated clearance of CD19+ tumor and prolongs the survival of tumor-bearing mice (Table 1; Qin et al., 2020). Interestingly, RNA-seq analysis reveals that only ibrutinib-treated, but not acalabrutinib-treated, CAR-T cells exhibit gene expression changes toward a memory-like T_H_1 phenotype, suggesting an ITK-dependent mechanism (Qin et al., 2020). Overall, the mechanisms of action for these two drugs on CAR-T cells are not clearly elucidated and represent an interesting area for future investigation. Despite of that, available evidence supports the therapeutic potential of combination or sequential therapies using ibrutinib or acalabrutinib and CAR-T cells.

## Natural Killer Cells and Natural Killer T Cells

Natural killer (NK) cells and natural killer T (NKT) cells are important effector cells of the innate immune system that contribute to immune responses against pathogens and tumor surveillance/immunity (Biron and Brossay, 2001; Woo et al., 2015). Similar to that observed for T cells, ibrutinib but not acalabrutinib inhibits AICD in *ex vivo* cultured human NK cells derived from healthy donors by reducing the upregulation of FASL in a dose-dependent manner (Long et al., 2017). However, the *in vivo* relevance of this finding is unclear. In CLL patients, ibrutinib treatment preserves circulating CD16+ NK cell counts but decreases immature CD16- NK cell counts and reduces the aberrantly elevated NKT cell counts at month 11 and stabilizes it thereafter (Solman et al., 2020, 2021). With acalabrutinib, circulating NK cell counts are briefly decreased at cycle 2 and then reverted back to baseline during subsequent cycles (Byrd et al., 2016). Therefore, these two BTK inhibitors exhibit different dynamic effects on circulating NK and NKT cell counts.

Bruton’s tyrosine kinase inhibitors are currently used and being tested in combination therapies with anti-CD20 and other antibodies for the treatment of B cell malignancies and other human diseases. As of May 2021, over 50 worldwide clinical trials registered on ClinicalTrials.gov are designed to assess the efficacy and safety of various antibody immunotherapy in combination with ibrutinib or acalabrutinib. NK cells play a vital role in cancer immunotherapies due to their expression of Fc receptors, which are activated by bound antibodies and mediate the killing of antibody-coated tumor or other target cells by NK cells. This defines a key mechanism of action for many therapeutic antibodies, termed ADCC. As mentioned above, one clinically significant difference between the impacts of the two BTK inhibitors on NK cell function is that ibrutinib but not acalabrutinib significantly affects ADCC (Table 1). It was found that in *in vitro* co-culture experiments, ibrutinib strongly inhibits healthy donor NK cell-mediated killing of CLL or MCL cells coated by anti-CD20 antibodies rituximab, ofatumumab or obinutuzumab (Bojarczuk et al., 2014; Kohrt et al., 2014; Da Roit et al., 2015; Hassenrück et al., 2018; Flinsenberg et al., 2019; Hofland et al., 2019). Continued oral ibrutinib treatment also inhibits anti-CD20 mediated activation of NK cells *in vivo* in CLL and MCL patients (Da Roit et al., 2015; Flinsenberg et al., 2019). Ibrutinib potently suppresses anti-CD20-induced calcium mobilization, IFNγ production, degranulation and cytotoxicity of NK cells, which all appear to be ITK-dependent, as these NK cell functions are not affected by more selective BTK inhibitors, including acalabrutinib, zanubrutinib or CGI-1746 (Kohrt et al., 2014; Da Roit et al., 2015; Hassenrück et al., 2018; Flinsenberg et al., 2019; Hofland et al., 2019). These results are in line with the observation that ITK is expressed in NK cells and regulates NK cell-mediated cytotoxicity and granule release (Khurana et al., 2007; Kohrt et al., 2014). ITK overexpression in NK cells results in enhanced FcR-initiated killing but reduced NKG2D-initiated cytotoxicity (Khurana et al., 2007). Overall, these studies reveal that the inhibitory effects of ibrutinib on ADCC can be attributed to its off-target inhibition of ITK in NK cells. Interestingly, a recent phase Ib/II study (NCT02296918) reported that acalabrutinib plus obinutuzumab (an anti-CD20 with enhanced ADCC activity) produce high and durable responses that deepen over time in CLL patients, while ibrutinib plus rituximab do not show benefits over the respective monotherapy (Woyach et al., 2020). Thus, understanding the differential effects of BTK inhibitors on ADCC will guide better design rationale in combination regimens involving these drugs and antibodies. When ibrutinib and antibody therapy are applied, appropriate sequential or alternate dosing schedules of ibrutinib *versus* antibody treatment episodes rather than concurrent administration should be considered.

Besides ITK, BTK also regulates NK cell function (Bao et al., 2012; Maffei et al., 2015). NK cells express moderate levels of BTK (Kohrt et al., 2014). Btk expression is upregulated during maturation and activation of mouse NK cells (Bao et al., 2012). *Btk*^–/–^ NK cells show reduced TLR3-induced NF-κB activation and immune responses, including IFNγ production, expression of perforin and granzyme B, and cytotoxicity (Bao et al., 2012). Poly(I:C)-induced NK cell-mediated acute hepatitis is attenuated in *Btk*^–/–^ mice or in mice received *in vivo* administration of a Btk inhibitor, LFM-A13 (Bao et al., 2012). NK cells derived from XLA patients with *BTK* mutations also exhibit decreased TLR3-induced activation, including IFNγ production, expression of CD69 and CD107a, and cytotoxicity. These findings indicate that BTK is required for TLR-induced NK cell activation (Bao et al., 2012; Maffei et al., 2015). However, information regarding the effects and clinical significance of ibrutinib and acalabrutinib on TLR-BTK-dependent NK cell activation in CLL and MCL patients is still lacking and awaits further investigation. Such information will also have implications for treatment-associated infections.

## Monocytes and Macrophages

In CLL patients, circulating monocyte counts are not changed by acalabrutinib treatment but are significantly and progressively increased by ibrutinib treatment toward healthy donor range (Byrd et al., 2016; Solman et al., 2020, 2021). Treatment with ibrutinib for 30 days reduces the refractory state of monocytes in CLL patients and restores lipopolysaccharide (LPS)-induced inflammatory responses through enhancing the phosphorylation of ERK1/2 and antigen presentation (Cubillos-Zapata et al., 2016). Long-term administration of ibrutinib is associated with enhanced HLA-DR expression on all monocyte subsets in CLL patients (Manukyan et al., 2018). Interestingly, ibrutinib treatment sustains the M2 phenotypes and immunosuppressive profile of nurse-like cells (NLCs), which are differentiated from monocytes and share the properties of M2-skewed tumor-associated macrophages (TAMs), in lymphoid organs of CLL patients by hampering TLR4 signaling (Tsukada et al., 2002; Filip et al., 2013; Boissard et al., 2015; Fiorcari et al., 2016). Ibrutinib treatment also alters M-CSF-induced differentiation of monocytes to fibrocyte-like cells with defective adhesion, impaired phagocytosis and enhanced production of reactive oxygen species (ROS) (Ferrarini et al., 2019). These findings are consistent with the notion that BTK regulates monocyte differentiation and macrophage polarization (Ni Gabhann et al., 2014). *Btk*^–/–^ macrophages show impaired LPS-induced M1 polarization but stronger tendency of M2 polarization, which is accompanied by reduced NF-κB activation and enhanced expression of the phosphatase SHIP1 (Ni Gabhann et al., 2014). Therefore, ibrutinib may induce M2 polarization and alter monocyte differentiation through BTK-dependent mechanisms. However, the detailed mechanisms and the effects of acalabrutinib on these processes await further investigation.

**FIGURE 1:**
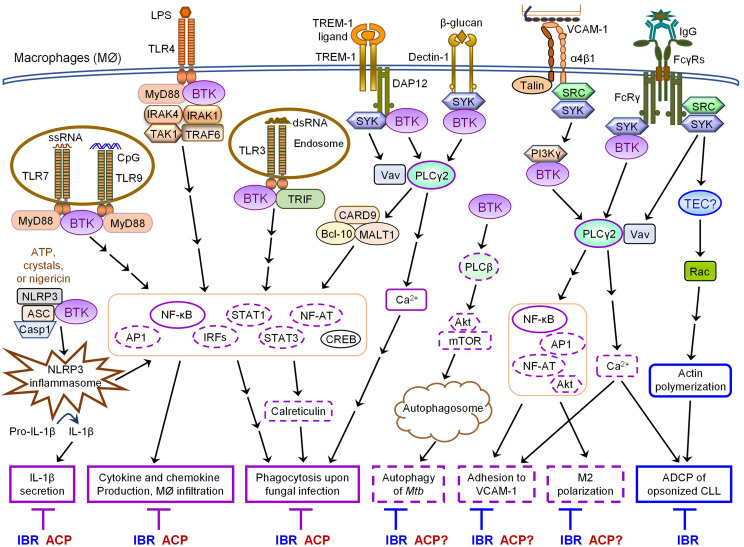
Overlapping and differential effects of ibrutinib and acalabrutinib on BTK-dependent *versus* -independent receptor signaling in macrophages. Major proximal signaling events and downstream effector functions of TLRs, NLRP3, TREM-1, Dectin-1, α4β1, and FcγRs in macrophages are illustrated in the figure. Both ibrutinib (IBR) and acalabrutinib (ACP) can inhibit TLR-, NLRP3-, TREM- 1-, and Dectin-1- induced signaling and effector functions (depicted in solid purple boxes in the figure) in macrophages via BTK-dependent mechanisms, suggesting the shared potential of both drugs in controlling certain inflammatory diseases and severe inflammation or cytokine storm associated with infections. Available evidence also demonstrates that ibrutinib inhibits α4β1- and FcγR-induced macrophage adhesion and M2 polarization as well as AKT-mTOR-induced autophagy via BTK-dependent mechanisms. These effects are predicted to be shared by acalabrutinib (depicted in dashed purple boxes in the figure), although direct evidence is still lacking and requires investigation. In contrast, ibrutinib but not acalabrutinib impairs FcγR-mediated ADCP of opsonized CLL cells by macrophages via BTK-independent mechanisms, likely through its off-target inhibition of TEC (depicted in solid blue boxes in the figure), suggesting differential potential of the two drugs in combination therapies with various depleting antibodies.

Both monocytes and macrophages express high levels of BTK (Rip et al., 2018). In these cells, BTK critically regulates TLR signaling by directly interacting with cytoplasmic Toll/IL-1 receptor (TIR) domains of most TLRs as well as their downstream adaptor proteins MYD88, TRIF, TIRAP and IRAK1 (Jefferies et al., 2003; Gray et al., 2006; Doyle et al., 2007; Liu et al., 2011; Lee et al., 2012; Marron et al., 2012; Chattopadhyay and Sen, 2014). Upon ligand binding, TLR-induced BTK phosphorylation promotes the activation of the transcription factors NF-κB and interferon-regulatory factors (IRFs) to enhance the expression of inflammatory cytokines, chemokines and interferons (IFNs) (Jefferies et al., 2003; Horwood et al., 2006; Doyle et al., 2007; Liljeroos et al., 2007; Liu et al., 2011; Lee et al., 2012; Marron et al., 2012; Chattopadhyay and Sen, 2014; Page et al., 2018). Monocytes or macrophages derived from XLA patients or *Btk*^–/–^ mice exhibit defective TNFα production in response to TLR2, 4, 7/8 signaling and IFN production in response to TLR3 signaling or viral infection (Jefferies et al., 2003; Horwood et al., 2006; Liu et al., 2011; Lee et al., 2012; Marron et al., 2012; Chattopadhyay and Sen, 2014; Page et al., 2018). In line with this, ibrutinib treatment inhibits LPS-induced production of CXCL12, CXCL13, CCL19 and VEGF in THP-1 macrophages (Ping et al., 2017). *In vivo* ibrutinib treatment leads to reduced serum levels of a variety of chemokines and inflammatory cytokines in CLL patients as well as decreased chemoattraction of CLL cells through inhibiting CXCL13 secretion by macrophages (Niemann et al., 2016). Acalabrutinib treatment similarly ameliorates LPS/galactosamine-induced infiltration of macrophages and reduces serum levels of MCP-1 in a mouse model of hepatic damage by inhibiting TLR4-induced NF-κB activation (Shaker et al., 2020). Interestingly, a number of case studies have reported the effects of ibrutinib or acalabrutinib on attenuating inflammatory cytokine and chemokine release, lung injury and respiratory failure in patients with severe COVID-19 (Lin et al., 2020; Rada et al., 2020; Scarfo et al., 2020; Treon et al., 2020; Alsuliman et al., 2021; Benner and Carson, 2021; Fiorcari et al., 2021; Molina-Cerrillo et al., 2021). Specifically, Roschewski et al. (2020) found that acalabrutinib treatment improves oxygenation and reduces IL-6 production in monocytes of patients with severe COVID-19. Furthermore, *BTK*-deficient or ibrutinib/acalabrutinib-treated monocytes and macrophages show defects in TLR-mediated phagocytosis of tumor cells as well as TLR9-, TREM-1 and Dectin-1-dependent production of inflammatory cytokines and phagocytosis upon fungal infection (Ormsby et al., 2011; Strijbis et al., 2013; Feng et al., 2015; Bercusson et al., 2018; Fiorcari et al., 2020). Monocytes and macrophages isolated from CLL patients treated with ibrutinib or acalabrutinib also exhibit reduced zymosan-induced phagocytosis (Fiorcari et al., 2020). Taken together, both ibrutinib and acalabrutinib can inhibit TLR-BTK, TREM-1-BTK and Dectin-1-BTK signaling pathways in monocytes and macrophages, resulting in reduced production of inflammatory cytokines and chemokines as well as impaired phagocytosis of tumor cells and infectious pathogens (Table 2 and Figure 1).

**TABLE 2:** Effects of ibrutinib and acalabrutinib on monocytes and macrophages.

Cells	Inhibitor	Target	Signaling pathway	Effects	References
Monocytes	Acalabrutinib	BTK	TLR9/TREM-1/Dectin-1-BTK	Inhibits TNF-α and IL-1β production and phagocytosis during *Aspergillus fumigatus* infection or stimulation with zymosan	Fiorcari et al., 2020
			TLR7/8-MyD88-BTK	Reduces IL-6 production in monocytes of COVID-19 patients	Roschewski et al., 2020
	Ibrutinib		Unclear	Progressively increases circulating monocyte counts in CLL patients	Solman et al., 2020, 2021
				Reduces the refractory state of monocytes in CLL patients	Cubillos-Zapata et al., 2016
				Restores LPS-induced inflammatory responses in monocytes of CLL patients	Cubillos-Zapata et al., 2016
				Enhances HLA-DR expression on monocytes in CLL patients after long-term administration	Manukyan et al., 2018
			FPR-BTK?	Inhibits chemoattractant-triggered inside-out signaling of β2 integrins (LFA-1 and Mac1) and thus adhesion to ICAM-1	Ferrarini et al., 2019
			LFA-1/Mac1-BTK?	Inhibits β2 integrin-mediated outside-in signaling and thus spreading on ICAM-1	Ferrarini et al., 2019
			M-CSFR-BTK/TEC?	Alters M-CSF-induced differentiation to fibrocyte- like cells with defective adhesion, impaired phagocytosis and enhanced ROS production	Ferrarini et al., 2019
		BTK	TLR9/TREM-1/Dectin-1-BTK	Inhibits TNF-α and IL-1β production and phagocytosis during *Aspergillus fumigatus* infection or stimulation with zymosan	Fiorcari et al., 2020
			NLRP3-BTK-PP2A	Enhances inflammasome activity in monocytes of CLL patients that received ibrutinib treatment	Mao et al., 2020
			α4β1-PI3Kγ-BTK-PLCγ2	Inhibits integrin activation and SDF1- or IL1β-mediated adhesion to VCAM1	Gunderson et al., 2016
			FcRγ-SYK-BTK-PLCγ2	Inhibits FcγR-mediated cytokine production	Ren et al., 2016
Macrophages	Acalabrutinib	BTK	TLR-MyD88-BTK-NF-κB	Suppresses LPS-induced MCP-1 production and macrophage infiltration	Shaker et al., 2020
			TLR9/TREM-1/Dectin-1-BTK	Inhibits TNF-α and IL-1β production and phagocytosis during *Aspergillus fumigatus* infection or stimulation with zymosan	Fiorcari et al., 2020
			TLR-NLPR3-BTK-NFκB	Reduces cytokine and chemokine production Attenuates sepsis-associated cardiac dysfunction in mice	O’Riordan et al., 2019
			None	Does not impair ADCP of rituximab-opsonized CLL cells	Golay et al., 2017; VanDerMeid et al., 2018
			FcγR-BTK?	Weakly inhibits ADCP of CLL cells opsonized with ofatumumab and ocaratuzumab	VanDerMeid et al., 2018
	Ibrutinib	BTK	TLR-MyD88-BTK-NF-κB/IRFs/STAT3/AP-1	Suppresses TLR-induced cytokine and chemokine production	Niemann et al., 2016; Ping et al., 2017
			TLR4-MyD88-BTK-STAT1	Sustains the M2 phenotypes and immunosuppressive profile of NLCs	Fiorcari et al., 2016
			TLR-BTK-calreticulin	Inhibits TLR-mediated phagocytosis of tumor cells	Feng et al., 2015
			TLR9-MyD88-BTK-NF-κB/NFATc1	Inhibits TNFα production and phagocytosis during fungal infection	Bercusson et al., 2018
			TLR9/TREM-1/Dectin-1- BTK	Inhibits TNF-α and IL-1β production and phagocytosis during *Aspergillus fumigatus* infection or stimulation with zymosan	Fiorcari et al., 2020
					
			NLRP3-ASC-BTK-NF-κB/caspase-1	Suppresses NLRP3-mediated inflammasome activation and blocks IL-1β processing	Ito et al., 2015; [Bibr B117][Bibr B10]
				Protects against ischemic brain injury by inhibiting NLRP3-mediated inflammasome activation	Ito et al., 2015
			NLRP3-BTK-PP2A	Enhances inflammasome activity by low doses of ibrutinib	Mao et al., 2020
			TLR-NLPR3-BTK-NF-κB	Reduces cytokine and chemokine production, affects bacterial clearance, and attenuates sepsis-associated cardiac dysfunction in mice	Benner et al., 2019; [Bibr B156][Bibr B117]; de Porto et al., 2019
			α4β1-PI3Kγ-BTK-PLCγ2	Inhibits integrin activation and SDF1- or IL1β-mediated adhesion to VCAM1	Gunderson et al., 2016
			BTK-PKCβ-AKT-mTOR-ATG/LC3b/p62	Induces autophagy of *M. tuberculosis* and suppresses Mtb intracellular growth	Hu et al., 2020
			FcRγ-SYK-BTK-PLCγ2	Promotes macrophage M1 polarization and inhibits macrophage M2 polarization	Gunderson et al., 2016
		TEC?	FcγR-TEC family?	Impairs ADCP of rituximab-opsonized CLL cells by human macrophages	Borge et al., 2015; Da Roit et al., 2015
					Golay et al., 2017; VanDerMeid et al., 2018
			FcγR-TEC/BTK?	Inhibits ADCP of CLL cells opsonized with ofatumumab and ocaratuzumab	VanDerMeid et al., 2018
		JAK2	FcγR-JAK2-STAT3/6	Enhances ADCP of opsonized MYC/BCL2 cells by mouse macrophage cell line J774A.1 cells	Barbarino et al., 2020

Notably, BTK is essential for NLRP3 inflammasome activation by physically interacting with NLRP3 and ASC (Ito et al., 2015; Liu et al., 2017; Song and Li, 2018; Weber, 2021). Upon NLRP3 activation, BTK is rapidly phosphorylated and in turn phosphorylates NLRP3 (Weber, 2021). Following priming by low-doses of LPS or polyI:C, *Btk*^–/–^ macrophages and PBMCs derived from XLA patients exhibit defective inflammasome activation, caspase-1 activation and IL-1β secretion in response to various activators of NLRP3 such as alum, crystals, ATP and nigericin, but show normal inflammasome activation and IL-1β secretion in response to the AIM2 activator Poly(dA:dT) (Ito et al., 2015; Liu et al., 2017; Weber, 2021). Corroborating the genetic evidence that BTK is a positive regulator of NLRP3 signaling, treatment with ibrutinib or acalabrutinib suppresses NLRP3- but not AIM2-induced inflammasome activation and blocks IL-1β processing in human and mouse macrophages or TAMs *in vitro* as well as *in vivo* in mouse models of ischemic brain injury, polymicrobial sepsis and bacterial infection (Ito et al., 2015; Liu et al., 2017; Benner et al., 2019; de Porto et al., 2019; O’Riordan et al., 2019; Weber, 2021). Paradoxically, following priming by a high-dose of LPS, *Btk*^–/–^ macrophages and monocytes derived from XLA patients display stronger NLRP3 inflammasome activation (Mao et al., 2020). Enhanced NLRP3 inflammasome activity is also observed in macrophages exposed to low doses of ibrutinib and in monocytes of CLL patients that received ibrutinib treatment (Mao et al., 2020). A negative role of BTK in NLRP3 inflammasome activation is also supported by the evidence that *Btk*^–/–^ mice exhibit increased severity of TNBS-induced colitis due to elevated IL-1β production, which may partially explain why XLA patients are prone to develop Crohn’s disease (Mao et al., 2020). However, such interpretation is confounded by the ablation of regulatory B cells (Bregs) in the BTK-deficient individuals and potential involvement of other inflammasomes in colitis (such as NLRC4, NLRP6, pyrin or AIM2) (Weber, 2021). Mechanistically, Mao et al. demonstrated that BTK binds to NLRP3 and inhibits the dephosphorylation of NLPR3 by the phosphatase PP2A to block the assembly of inflammasome (Mao et al., 2020). Thus, the exact effects of BTK, ibrutinib and acalabrutinib on NLRP3 inflammasome activation appear to be much more complex than initially revealed and may vary drastically depending on the concentrations of different stimuli or drugs and the extent or stage of inflammasome assembly in different cellular contexts.

Bruton’s tyrosine kinase also participates in the regulation of adhesion and autophagy in monocytes and macrophages (Gunderson et al., 2016; Ferrarini et al., 2019; Hu et al., 2020). An integrin α4β1-PI3Kγ-BTK-PLCγ2 signaling pathway is activated in macrophages prior to cell adhesion to VCAM1 (Gunderson et al., 2016). Ibrutinib has been shown to inhibit α4β1 integrin-mediated adhesion of macrophages to VCAM1 and disaggregate the interactions between macrophages and CLL cells in the bone marrow (Gunderson et al., 2016; Pleyer et al., 2018). Ibrutinib also affects both chemoattractant-triggered inside-out signaling of β2 integrins (LFA-1 and Mac1) and β2 integrin-mediated outside-in signaling events, thus inhibiting monocyte adhesion and spreading on immobilized ICAM-1 (Ferrarini et al., 2019), although the detailed mechanism of action is unclear. Interestingly, ibrutinib suppresses *Mycobacterium tuberculosis* (*Mtb*) survival in human macrophages by inducing complete autophagy flux through inhibition of the BTK-AKT-mTOR pathway (Hu et al., 2020). *In vivo* administration of ibrutinib reduces *Mtb* load in the mediastinal lymph nodes and spleens of *Mtb*-infected mice, suggesting that ibrutinib may serve as a promising anti-TB drug (Hu et al., 2020). It is likely that acalabrutinib can also inhibit BTK-dependent adhesion and autophagy, but this remains to be directly tested in monocytes and macrophages.

The most important difference between the effects of ibrutinib and acalabrutinib on monocytes and macrophages is on FcγR-mediated antibody-dependent cellular phagocytosis (ADCP), one of the major mechanisms for clearance of opsonized malignant B cells in anti-CD20-based therapies (Maffei et al., 2015; VanDerMeid et al., 2018). Early evidence showed that BTK is activated by FcγR and is required for FcγR-mediated optimal phagocytosis (Jongstra-Bilen et al., 2008). However, ibrutinib treatment does not impair FcγR-mediated phagocytosis of opsonized sheep red blood cells, but suppresses FcγR-induced cytokine production such as TNFα in *in vitro* cultured human peripheral blood monocytes and inhibits FcγR-mediated M2 polarization of macrophages (Gunderson et al., 2016; Ren et al., 2016). Notably, ibrutinib but not acalabrutinib significantly impairs ADCP of rituximab-opsonized CLL cells by human macrophages, suggesting a BTK-independent mechanism of action (Borge et al., 2015; Da Roit et al., 2015; Golay et al., 2017). Acalabrutinib has also been shown to be less inhibitory than ibrutinib on ADCP of CLL cells opsonized with the next-generation anti-CD20 mAbs ofatumumab and ocaratuzumab by human macrophages (VanDerMeid et al., 2018). In line with this, ibrutinib in combination with rituximab or obinutuzumab do not show favorable clinical outcomes compared to ibrutinib monotherapy in clinical studies, which may be attributed to impaired ADCC and ADCP mechanisms (Rogers and Woyach, 2020). On the contrary, however, one recent study has reported that ibrutinib, but not acalabrutinib or other second-generation BTK inhibitors, enhances ADCP of human MYC/BCL-2 cell line opsonized with mAbs (rituximab, obinutuzumab or alemtuzumab) by mouse macrophage cell line J774A.1 through off-target inhibition of JAK2-STAT3/STAT6 signaling (Barbarino et al., 2020). The contradictory effects of ibrutinib on ADCP observed in different studies may be attributable to the use of different macrophages and target cells. Despite of that, all these studies suggest that ibrutinib affects ADCP via BTK-independent, off-target inhibition of other kinases such as TEC and JAK2, while acalabrutinib does not significantly interfere with ADCP (Jongstra-Bilen et al., 2008; Borge et al., 2015; Da Roit et al., 2015; Golay et al., 2017; VanDerMeid et al., 2018; Barbarino et al., 2020).

Collectively, both ibrutinib and acalabrutinib can inhibit TLR-, TREM- 1-, Dectin-1- and NLRP3-induced signaling pathways and effector functions in monocytes and macrophages via BTK-dependent mechanisms (Figure 1 and Table 2), suggesting the shared potential of both drugs in controlling certain inflammatory diseases and severe inflammation or cytokine storm associated with infections. In contrast, ibrutinib but not acalabrutinib impairs FcγR-mediated ADCP of opsonized CLL cells by human macrophages likely via off-target inhibition of TEC (Figure 1 and Table 2), suggesting differential potential of these two BTK inhibitors in combination therapies with various depleting antibodies.

## Granulocytes and Mast Cells

It is intriguing that acalabrutinib treatment improves neutrophil counts in most relapsed CLL patients that had cytopenia at baseline (Byrd et al., 2016), while no effect of ibrutinib treatment on circulating neutrophil counts has been reported for CLL and MCL patients. Given the evidence that neutrophils from patients with XLA are more susceptible to apoptosis due to ROS accumulation (Honda et al., 2012), it is likely that acalabrutinib improves neutrophil counts through an indirect immunomodulatory mechanism. Occasional cases of neutrophilic panniculitis, characterized by predominantly neutrophilic inflammation in the subcutaneous fat, has been reported as an emerging adverse reaction in CLL patients that received ibrutinib therapy (Stewart et al., 2018), although the underlying mechanism is unclear.

Similar to that observed in monocytes and macrophages, ibrutinib inhibits TLR-, TREM-1- and NLRP3-induced inflammatory responses and effector functions via BTK-dependent mechanisms in granulocytes (Table 3). Ibrutinib treatment reduces TLR2-induced CD11b expression on neutrophils upon *in vitro* stimulation with lipoteichoic acid (LTA) and *in vivo* neutrophil influx in the lung in response to intranasal LTA instillation (de Porto et al., 2019). Ibrutinib treatment also attenuates TLR7-induced Btk phosphorylation, oxidative stress and production of IL-23 and TNFα in dermal and splenic neutrophils in a mouse model of imiquimod-induced psoriatic inflammation (Al-Harbi et al., 2020). Interestingly, ibrutinib significantly inhibits TREM-1-induced oxidative burst and completely abrogates TREM-1-induced ERK1/2 phosphorylation, CD62L shedding and CD66b upregulation in human neutrophils purified from healthy donors, but only partially inhibits LPS-induced CD66b upregulation and does not inhibit LPS-induced ERK1/2 phosphorylation or CD62L shedding (Stadler et al., 2017). Ibrutinib also suppresses NLRP3 inflammasome activation and caspase-1 activity, thus blocking maturation of IL-1β in infiltrating neutrophils in a mouse model of ischemic brain injury (Ito et al., 2015).

**TABLE 3:** Effects of ibrutinib and acalabrutinib on granulocytes and mast cells.

Cells	Inhibitor	Target	Signaling pathway	Effects	References
Granulocytes	Acalabrutinib		Unclear	Improves neutrophil counts in most relapsed CLL patients that had cytopenia at baseline	Byrd et al., 2016
		BTK	FcεR-BTK	Inhibits IgE-induced degranulation, release of histamine, leukotriene C4 and IL-4, and upregulation of CD63, CD164, CD203c or LAMP1 in basophils	Dispenza et al., 2020
	Ibrutinib		Unclear	Induces occasional cases of neutrophilic inflammation in the subcutaneous fat in CLL patients	Stewart et al., 2018
		BTK	TLR2-MYD88-BTK	Inhibits LTA-induced neutrophil influx in the lung and reduces LTA-induced CD11b expression on neutrophils	de Porto et al., 2019
			TLR4-MYD88-BTK	Partially inhibits LPS-induced CD66b upregulation in neutrophils	Stadler et al., 2017
			TLR7-MYD88-BTK	Attenuates imiquimod-induced oxidative stress and production of IL-23 and TNFα in neutrophils	Al-Harbi et al., 2020
			TREM-1-BTK-ERK1/2	Inhibits neutrophil oxidative burst, CD62L shedding and CD66b upregulation	Stadler et al., 2017
			NLRP3-ASC-BTK-NF-κB/caspase-1	Suppresses NLRP3-induced inflammasome activation and IL-1β processing in neutrophils	Ito et al., 2015
			TLR/TREM-1/NLRP3-BTK	Inhibits *E. coli*-induced oxidative burst, bacteria killing capacity and NET production of neutrophils	Prezzo et al., 2019; Risnik et al., 2020
				Reduces systemic neutrophil activation and neutrophil influx in the lung during ceftriaxone- treated pneumococcal pneumonia in mice	de Porto et al., 2019
				Impairs ROS production, phagocytosis and microbicidal capacity of neutrophils in response	Stadler et al., 2017; Blez et al., 2020
			TLR/CLR-BTK?	Attenuates neutrophilic and eosinophilic inflammation induced by cockroach allergens	Nadeem et al., 2019
			FcεR-BTK	Inhibits IgE-induced degranulation, release of histamine, leukotriene C4 and IL-4, and upregulation of CD63, CD164, CD203c or LAMP1 in basophils	MacGlashan et al., 2011 Smiljkovic et al., 2017 Dispenza et al., 2020
				Eliminates IgE-mediated basophil activation test response to aeroallergens in CLL patients	Dispenza et al., 2017; Regan et al., 2017
		TEC?	FcγR-TEC family?	Decreases IL-8 production, degranulation and release of NE, MPO and lactoferrin by neutrophils in response to opsonized E. coli	Prezzo et al., 2019
				Inhibits ADCP of lymphoma cells opsonized with anti-CD20 or tumor cells opsonized with	Da Roit et al., 2015; Duong et al., 2015
Mast cells	Acalabrutinib	BTK	FcεR-BTK	Inhibits IgE-induced degranulation, release of histamine, leukotriene C4 and IL-4, and upregulation of CD63, CD164, CD203c or LAMP1 in mast cells	Dispenza et al., 2020
					Smiljkovic et al., 2017
				Prevents allergen-IgE-mediated broncho-constriction in isolated human lung tissues	Dispenza et al., 2020
				Protects against IgE-mediated systemic anaphylaxis and death	Dispenza et al., 2020
	Ibrutinib	BTK	FcεR-BTK	Inhibits IgE-induced degranulation, release of histamine, leukotriene C4 and IL-4, and upregulation of CD63, CD164, CD203c or LAMP1 in mast cells	Dispenza et al., 2020
				Prevents allergen-IgE-mediated broncho-constriction in isolated human lung tissues	Dispenza et al., 2020
				Eliminates skin prick test reactivity in CLL patients	Dispenza et al., 2017; Regan et al., 2017

In line with its effects on receptor signaling pathways, ibrutinib treatment generally affects neutrophil activation in response to bacterial or fungal infection, which typically engage multiple TLRs, TREM-1 and NLRP3. Neutrophils purified from ibrutinib-treated CLL patients exhibit reduced *E. coli*-induced oxidative burst and bacteria killing capacity and slightly impaired neutrophil extracellular trap (NET) production (Prezzo et al., 2019; Risnik et al., 2020). Ibrutinib treatment reduces systemic neutrophil activation and neutrophil influx in the lung during ceftriaxone-treated pneumococcal pneumonia in mice (de Porto et al., 2019). Both ibrutinib-treated neutrophils obtained from healthy donors and neutrophils from ibrutinib-treated CLL patients show decreased ROS production as well as impaired phagocytosis and microbicidal capacity in response to infection by *Aspergillus fumigatus* (Blez et al., 2020). Ibrutinib treatment also inhibits *in vivo* neutrophil activation, resulting in increased fungal burden in the lung in a mouse model of fungal infection by *A. fumigatus* conidia (Stadler et al., 2017). Furthermore, ibrutinib treatment potently suppresses Btk phosphorylation in neutrophils and attenuates neutrophilic and eosinophilic inflammation in a mouse model of cockroach allergen extract (CAE)-induced eosinophilic and neutrophilic asthma, which involves the activation of TLRs, CLRs and PAR-2 in granulocytes (Gao, 2012; Nadeem et al., 2019). However, information regarding the effects of acalabrutinib in these BTK-dependent signaling pathways in neutrophils is still lacking and awaits investigation.

Ibrutinib also inhibits FcγR signaling in neutrophils, causing reduced neutrophil degranulation and ADCP in response to opsonized pathogens and malignant cells. In the early phases of treatment, neutrophils purified from ibrutinib-treated CLL patients display decreased FcγR-mediated IL-8 production and degranulation in response to opsonized *E. coli*, leading to reduced release of neutrophil elastase (NE), myeloperoxidase (MPO) and lactoferrin (Prezzo et al., 2019). Ibrutinib potently inhibits ADCP of lymphoma cells opsonized with anti-CD20 (rituximab, obinutuzumab or ofatumumab) or tumor cells opsonized with anti-HER2 (trastuzumab) by fresh human neutrophils *in vitro* (Da Roit et al., 2015; Duong et al., 2015). Interestingly, neutrophils of XLA patients exhibit normal IL-8 production, degranulation and ADCP in response to opsonized bacteria and malignant cells (Cavaliere et al., 2017). Given the lack of effects of acalabrutinib on FcγR-mediated ADCP in macrophages (Golay et al., 2017; VanDerMeid et al., 2018), it is not likely that acalabrutinib could inhibit FcγR-mediated ADCP in neutrophils, although direct evidence has not been reported. Thus, the inhibitory effects of ibrutinib on FcγR-mediated neutrophil activation and ADCP are likely mediated through its off-target inhibition on non-BTK TEC family kinases that modulate neutrophil functions (Prezzo et al., 2019). The exact target kinases and detailed molecular mechanisms of ibrutinib on FcγR signaling in neutrophils remain to be elucidated in future studies.

Of particular relevance to the prevention and treatment of allergy, both ibrutinib and acalabrutinib can inhibit FcεR-BTK-mediated signaling and allergic responses in basophils and mast cells. Treatment with ibrutinib or acalabrutinib abolishes IgE-induced degranulation and release of histamine, leukotriene C4 (LTC4) and IL-4 as well as upregulation of activation markers CD63, CD164, CD203c or LAMP1 in human basophils and mast cells *in vitro* (MacGlashan et al., 2011; Smiljkovic et al., 2017; Dispenza et al., 2020). Treatment with ibrutinib also eliminates skin prick test reactivity and IgE-mediated basophil activation test (BAT) responses to aeroallergens in CLL patients (Dispenza et al., 2017; Regan et al., 2017). The role of BTK in this pathway has been revealed by the evidence that bone marrow-derived mast cells of *Btk*^–/–^ mice exhibit impaired FcεRI-mediated production of eicosanoid, LTC4 and ROS *in vitro* (Kuehn et al., 2008). Interestingly, ibrutinib or acalabrutinib prevents allergen-IgE-mediated bronchoconstriction in isolated human lung tissues *ex vivo* and acalabrutinib effectively protects against IgE-mediated systemic anaphylaxis and death in a humanized mouse model *in vivo*, suggesting an almost complete blockade of histamine and leukotriene release by mast cells and basophils (Dispenza et al., 2020). Therefore, by acting on TLR, TREM-1, NLRP3, FcγR and FcεR signaling pathways, ibrutinib and acalabrutinib may compromise innate immune responses of granulocytes against bacterial and fungal infections, but may also provide protection against damaging inflammatory responses of neutrophils and eosinophils as well as allergic responses of basophils and mast cells (Table 3).

## Myeloid-Derived Suppressor Cells

Myeloid-derived suppressor cells (MDSCs) potently suppress both adaptive and innate immune responses and are recognized barriers of cancer immunotherapy (Veglia et al., 2018). Ibrutinib treatment significantly decreases the aberrantly elevated counts of MDSCs and effectively normalizes MDSC counts to healthy donor range within the first 1–2 years of therapy in CLL patients, suggesting that ibrutinib continuously improves the immunosuppressive condition in these patients (Solman et al., 2020, 2021). Interestingly, a recent phase II clinical trial on patients with advanced pancreatic cancer has demonstrated that acalabrutinib monotherapy or combination therapy with the anti-PD-1 antibody pembrolizumab leads to consistent and durable reductions in peripheral blood granulocytic (CD15+) MDSCs over time, with median reduction of >50% achieved after 2–3 weeks of therapy (Overman et al., 2020). In another phase II clinical trial on patients with platinum-refractory metastatic urothelial carcinoma, acalabrutinib plus pembrolizumab therapy also led to decreased MDSC levels in a patient with high MDSCs at the baseline (Zhang et al., 2020). These findings suggest that both ibrutinib and acalabrutinib may help to control the abnormal expansion of MDSCs and thus relieve immunosuppression in cancer patients.

Human and murine MDSCs express BTK (Stiff et al., 2016; Ishfaq et al., 2021). Increased expression of BTK correlates with a poor relapse-free survival probability in patients with neuroblastoma (Ishfaq et al., 2021). Similar to that observed in CLL patients, ibrutinib treatment results in a significant reduction of MDSCs in the spleen and tumor in WT mice transplanted with mammary tumors, melanomas or neuroblastomas, but not in transplanted XID mice harboring a BTK mutation, suggesting a BTK-dependent mechanism of action for ibrutinib on MDSCs (Stiff et al., 2016; Varikuti et al., 2020; Ishfaq et al., 2021). Reduced MDSCs in these tumor-bearing WT mice are accompanied by increased T cell infiltration and effector functions as well as decreased tumor growth and metastasis (Stiff et al., 2016; Varikuti et al., 2020; Ishfaq et al., 2021). Varikuti et al. (2020) revealed that ibrutinib-mediated reduction of MDSCs is associated with increased frequency of mature DCs in the spleen and tumor of transplanted WT mice and that *ex vivo* treatment of MDSCs with ibrutinib induces their maturation toward CD11c+MHCII+ DCs. Interestingly, ibrutinib inhibits *in vitro* generation of human MDSCs from healthy donor monocytes induced by GM-CSF and IL-6, and also inhibits the phosphorylation of BTK and significantly reduces the expression of the immunosuppressive gene *Ido1* in the *in vitro* generated human MDSCs (Stiff et al., 2016). Ibrutinib also inhibits LPS-induced phosphorylation of BTK, expression of *Arg1, Nos2*, *Ido1* and *Tgfb*, and production of TNFα and NO in the murine MDSC cell line MSC2 and primary MDSCs isolated from mice bearing neuroblastoma (Stiff et al., 2016). Ibrutinib-treated MDSCs display defects in suppressing T cell proliferation and activation *in vitro* (Ishfaq et al., 2021). In addition, ibrutinib reduces the expression of adhesion molecules CD49D and CD11a on MDSCs, which are known to play a role in MDSC migration (Stiff et al., 2016). Indeed, ibrutinib impairs the *in vitro* migration of MSC2 cells induced by cancer cell-conditioned media and human MDSCs induced by GM-CSF or CXCL12 (also known as SDF-1), and also restricts the *in vivo* migration of MDSCs into the tumor microenvironment (TME) in melanoma-bearing mice (Stiff et al., 2016; Conniot et al., 2019). However, the effects of acalabrutinib on MDSC generation, immunosuppressive function or migration have not been reported yet. Overall, the information of the effects of ibrutinib and acalabrutinib on MDSCs is very limited (Table 4) and their mechanisms of action in MDSCs remain elusive, representing an interesting and significant area for future research.

**TABLE 4:** Effects of ibrutinib and acalabrutinib on MDSCs, DCs and osteoclasts.

Cells	Inhibitor	Target	Signaling pathway	Effects	References
MDSCs	Acalabrutinib	BTK	Unclear	Reduces peripheral blood granulocytic MDSCs in patients with advanced pancreatic cancer and metastatic urothelial carcinoma	Overman et al., 2020; Zhang et al., 2020
	Ibrutinib	BTK	Unclear	Decreases the elevated counts of MDSCs in CLL patients	Solman et al., 2020, 2021
				Reduces MDSCs in the spleen and tumor in mice bearing transplanted solid tumors	Stiff et al., 2016; Varikuti et al., 2020
					Ishfaq et al., 2021
				Induces the maturation of MDSCs toward CD11c+MHCII+ DCs *in vitro* and in mice bearing transplanted E0771 mammary tumors	Varikuti et al., 2020
				Inhibits MDSC-mediated suppression of T-cell proliferation and activation	Stiff et al., 2016; Ishfaq et al., 2021
				Reduces the expression of adhesion molecules CD49D and CD11a on MDSCs	Stiff et al., 2016
			GM-CSFR/IL-6R-BTK	Inhibits GM-CSF+IL-6-induced *in vitro* generation of MDSCs from normal human blood monocytes	Stiff et al., 2016
				Reduces GM-CSF+IL-6-induced *Ido1* expression in *in vitro* generated MDSCs	Stiff et al., 2016
			TLR4-MyD88-BTK	Inhibits LPS-induced expression of *Arg1*, *Nos2*, Ido1 and Tgfb as well as production of TNFα and NO in MDSCs	Stiff et al., 2016
			GM-CSFR-BTK	Impairs GM-CSF-induced migration of MDSCs	Stiff et al., 2016
			CXCR4-BTK	Inhibits CXCL12-induced *in vitro* migration and *in vivo* migration of MDSCs into the TME	Stiff et al., 2016; Conniot et al., 2019
DCs	Ibrutinib		Unclear	Gradually increases the counts of plasmacytoid DCs in CLL patients	Solman et al., 2021
		BTK	GM-CSFR-BTK	Accelerates GM-CSF-induced maturation, down-regulates the expression of Ly6C and up-regulates MHC class II and CD80 in DCs	Natarajan et al., 2016a
			TLR4-MyD88-BTK	Decreases LPS-induced up-regulation of CD86 and production of IL-6, IL-12 and NO in differentiating BMDCs	Natarajan et al., 2016a
				Increases LPS-induced upregulation of MHC class II, CD80 and CCR7, production of IFNβ and IL-10, and the ability to activate CD4 T cells in differentiating BMDCs	Natarajan et al., 2016a
				Inhibits LPS-induced production of TNFα and NO as well as expression of MHC class II and CD86 in differentiated BMDCs	Natarajan et al., 2016a
				Elevates LPS-induced up-regulation of CD80, production of IL-6, IL-18, IL-10 and TGFβ,and the ability to drive Th17 response in differentiated BMDCs	Natarajan et al., 2016a
			TLR7-MYD88-BTK	Attenuates imiquimod-induced oxidative stress and production of IL-23 and TNFα in dermal and splenic DCs	Al-Harbi et al., 2020; Nadeem et al., 2020
			TLR9-MYD88-BTK-STAT3/STAT1	Impairs CpG-induced up-regulation of CD86, CD83, CD80 and HLA-DR as well as production of IL-6, IL-12, TNFα and IL-10	Lougaris et al., 2014
Osteoclasts	Acalabrutinib	BTK	RANK-BTK-PLCγ1/γ2-NFATc1/c-Fos/NF-κB	Inhibits RANKL-induced osteoclast differentiation from monocytes or macrophages	Pokhrel et al., 2019; Liu et al., 2021
				Reduces the bone-resorbing activities of osteoclasts induced by RANKL and M-CSF	Pokhrel et al., 2019
				Ameliorates bone damage and arthritis severity in a mouse model of collagen-induced arthritis	Liu et al., 2021
			TLR4-MyD88-BTK-NFATc1/c-Fos	Inhibits LPS-induced osteoclast differentiation from RANKL-primed osteoclast precursors	Pokhrel et al., 2019
				Protects against *Porphyromonas gingivalis* LPS- induced alveolar bone erosion in a mouse model of periodontitis	Pokhrel et al., 2019
	Ibrutinib	BTK	RANK-BTK-PLCγ1/γ2-NFATc1/SRC	Inhibits RANKL-induced osteoclast differentiation from monocytes or macrophages	Shinohara et al., 2014; Liu et al., 2021
				Reduces the bone-resorbing activities of osteoclasts induced by RANKL and M-CSF	Shinohara et al., 2014
				Protects against bone loss in a mouse model of RANKL-induced osteoporosis	Shinohara et al., 2014
				Ameliorates bone damage and arthritis severity in a mouse model of collagen-induced arthritis	Liu et al., 2021

## Dendritic Cells

Dendritic cells (DCs) are the most potent antigen-presenting cells, linking the innate arm of the immune response to the adaptive counterpart (Chudnovskiy et al., 2019; Heath et al., 2019). While no effect of acalabrutinib on DC counts has been reported, ibrutinib treatment gradually increases the counts of plasmacytoid DCs in CLL patients from abnormally low at baseline to healthy donor range at 2 years after treatment (Solman et al., 2021).

Mechanistically, ibrutinib promotes DC maturation and differentially affects DC activation via BTK-dependent pathways. BTK is expressed in DCs (Kawakami et al., 2006). In response to LPS-induced TLR4 signaling, *in vitro* cultured bone marrow-derived DCs (BMDCs) of *Btk*^–/–^ mice exhibit enhanced maturation and increased up-regulation of the co-stimulatory molecules CD80 and CD86, but decreased production of the anti-inflammatory cytokine IL-10 (Kawakami et al., 2006). Similarly, ibrutinib treatment accelerates GM-CSF-induced maturation, augments the up-regulation of MHC class II and CD80, and down-regulates the expression of Ly6C in DCs derived from WT mice (Natarajan et al., 2016a). *Btk*^–/–^ DCs display an enhanced *in vivo* activity at stimulating IgE response, T_H_2-driven asthma and T_H_1-driven contact sensitivity in mouse models (Kawakami et al., 2006). BMDCs differentiated under ibrutinib treatment subsequently show altered responses to LPS stimulation, including increased upregulation of MHC class II, CD80 and CCR7, increased production of IFNβ and IL-10, and enhanced ability to activate CD4 T cells in co-culture experiments, but decreased up-regulation of CD86 and reduced production of IL-6, IL-12 and nitric oxide (NO) (Natarajan et al., 2016a). When ibrutinib treatment is applied after the differentiation of BMDCs is completed, it exerts different effects on LPS-induced activation of BMDCs, including elevated up-regulation of CD80, increased production of IL-6, IL-18, IL-10 and TGFβ, and enhanced ability to drive T_H_17 response in co-culture experiments, but decreased production of TNFα and NO as well as dampened expression of MHC class II and CD86 (Natarajan et al., 2016b). As observed in neutrophils, ibrutinib also attenuates TLR7-induced Btk phosphorylation, oxidative stress and production of inflammatory cytokines IL-23 and TNFα in dermal and splenic DCs in a mouse model of imiquimod-induced psoriatic inflammation (Al-Harbi et al., 2020; Nadeem et al., 2020). Furthermore, ibrutinib treatment impairs CpG-, but not LPS-, induced activation of STAT1/STAT3 and up-regulation of CD86, CD83, CD80 and HLA-DR as well as production of cytokines IL-6, IL-12 and TNFα in human monocyte-derived DCs prepared from healthy donors by inhibiting TLR9 signaling (Lougaris et al., 2014). A BTK-dependent mechanism of action has been verified by similarly impaired CpG-, but not LPS-, induced activation of DCs derived from XLA patients (Lougaris et al., 2014). Taken together, ibrutinib treatment modulates the maturation and activation of DCs induced by GM-CSF, TLR4, TLR7 and TLR9 signaling via BTK-dependent mechanisms (Table 4). Acalabrutinib is predicted to have similar effects on DCs, but direct evidence is still lacking.

## Osteoclasts

Bone-resorbing osteoclasts play an essential role in normal bone homeostasis. Dysregulation of osteoclasts has been implicated in the pathogenesis of several bone disorders such as osteoporosis, periodontitis and rheumatoid arthritis (RA) (Takayanagi, 2007). BTK is expressed in osteoclasts and is essential for RANKL-induced osteoclast differentiation (Lee et al., 2008). Both ibrutinib and acalabrutinib potently inhibit RANKL-induced osteoclast differentiation in *in vitro* cultured bone marrow-derived monocytes/macrophages (BMMs) or RAW264.7 cells via the RANK-BTK-PLCγ1/γ2-NF-ATc1 pathway (Shinohara et al., 2014; Pokhrel et al., 2019; Liu et al., 2021). Acalabrutinib also inhibits LPS-induced osteoclast differentiation from RANKL-primed osteoclast precursors via the TLR4-BTK-NF-ATc1/c-Fos pathway (Pokhrel et al., 2019). Both ibrutinib and acalabrutinib can significantly reduce the bone-resorbing activities of osteoclasts following treatment with RANKL and M-CSF (Shinohara et al., 2014; Pokhrel et al., 2019). Interestingly, reduction in resorption activities of osteoblasts by ibrutinib is mediated through suppression of the expression of Src, Ptk2, Ptk2b and Talin 1 via an NF-ATc1-independent mechanism (Shinohara et al., 2014). In line with the *in vitro* evidence, *in vivo* administration of ibrutinib protects against bone loss in a mouse model of RANKL-induced osteoporosis (Shinohara et al., 2014). Similarly, acalabrutinib treatment protects against *Porphyromonas gingivalis* LPS-induced alveolar bone erosion in a mouse model of periodontitis (Pokhrel et al., 2019). Furthermore, oral administration of ibrutinib or acalabrutinib ameliorates bone damage and arthritis severity in a mouse model of collagen-induced arthritis (Table 4; Liu et al., 2021). Together, these findings suggest that BTK inhibitors are new therapeutic candidates for the treatment of bone disorders involving bone destruction. On the other hand, long-term treatment with ibrutinib or acalabrutinib may affect bone homoeostasis due to inhibition of osteoclast differentiation and function.

## Megakaryocytes and Platelets

One of the most common adverse effects of ibrutinib and acalabrutinib is an increased risk of bleeding in CLL and MCL patients (Busygina et al., 2019). Approximately 30–50% of CLL and MCL patients treated with ibrutinib or acalabrutinib have low-grade (grade 1–2) bleeding. However, ibrutinib treatment is associated with an increased risk of major bleeding (grade 3–4), which is much reduced with acalabrutinib treatment (Byrd et al., 2016; Wang et al., 2018; Pellegrini et al., 2021). After initiation of ibrutinib therapy, the majority of CLL patients show a small decrease in platelet counts on day 2, which is followed by a rapid increase in platelet counts several days later (Lipsky et al., 2015; Huang et al., 2021). Platelets from ibrutinib-treated CLL patients exhibit reduced surface levels of GPIb-IX complex and αIIbβ3 integrin (also known as GPIIb/IIIa), higher membrane fluidity, lower resting membrane potential and higher level of ROS production compared to those derived from untreated CLL patients and healthy volunteers (Dobie et al., 2019; Popov et al., 2020). *In vitro* treatment of whole blood from healthy donors with ibrutinib induces a time-dependent shedding of GPIb-IX complex and αIIbβ3 integrin from the platelet surface by activating ADAM17 and an unknown sheddase (Dobie et al., 2019). These findings suggest that ibrutinib has complex effects on platelet counts and platelet physiology in patients.

Consistent with the clinical observation, ibrutinib treatment impairs the proliferation of megakaryocyte progenitor cells during early stage megakaryopoiesis and decreases the number of colony-forming units of megakaryocytes (CFU-MKs) derived from human cord blood CD34+ hematopoietic stem cells (HSCs) or a human megakaryoblastic cell line SET-2 *in vitro* (Huang et al., 2021). On the other hand, ibrutinib enhances the differentiation and ploidy of megakaryocytes as well as proplatelet formation during late-stage megakaryopoiesis (Huang et al., 2021). Ibrutinib also impairs megakaryocyte adhesion and spreading on immobilized fibrinogen by inhibiting the integrin αIIbβ3 outside-in signaling in megakaryocytes (Huang et al., 2021). *In vivo* administration of ibrutinib in C57BL/6 mice results in thrombocytopenia in the bone marrow associated with a decrease in platelet counts at day 2 to day 7, which is recovered to normal levels by day 15 after treatment (Huang et al., 2021). Thus, the complex effects of ibrutinib on platelet counts are mediated at least partially by its differential modulation of megakaryocyte differentiation and function at different stages of megakaryopoiesis (Table 5), although the underlying molecular mechanisms remain unclear and require further investigation.

**TABLE 5:** Effects of ibrutinib and acalabrutinib on megakaryocytes and platelets.

Cells	Inhibitor	Target	Signaling pathway	Effects	References
Megakaryocytes	Ibrutinib		Unclear	Impairs the proliferation of progenitor cells during early stage megakaryopoiesis	Huang et al., 2021
				Decreases the number of colony-forming units of megakaryocytes (CFU-MKs) derived from HSCs	Huang et al., 2021
				Enhances the differentiation and ploidy of megakaryocytes and the formation of proplatelets during late-stage megakaryopoiesis	Huang et al., 2021
				Increases the expression of integrin αIIbβ3 on megakaryocytes	Huang et al., 2021
				Induces thrombocytopenia in the bone marrow of mice	Huang et al., 2021
		SRC/BTK	αIIbβ3-SRC-SYK-BTK-PLCγ2-AKT/ERK1/2	Impairs megakaryocyte adhesion and spreading on immobilized fibrinogen	Huang et al., 2021
				Inhibits the integrin αIIbβ3 outside-in signaling in megakaryocytes	Huang et al., 2021
Platelets	Acalabrutinib IC_50_ 1.85 μM	BTK	αIIbβ3-SYK-PI3K-BTK-PLCγ2	Inhibits platelet spreading on fibrinogen, cytoskeletal assembly and platelet aggregation	Zheng et al., 2021
			GPIb-IX-SYK-LAT-PI3K-BTK-PLCγ2	Inhibits ristocetin- or VWF-induced platelet adhesion and aggregation	Denzinger et al., 2019
			CLEC-2-SYK-LAT-BTK-PLCγ2-NFAT	Blocks CLEC-2-mediated platelet activation and granule secretion	Nicolson et al., 2021
				Platelets from acalabrutinib-treated CLL patients do not aggregate in response to rhodocytin or podoplanin	Nicolson et al., 2021
			FcγRIIA-BTK-PLCγ2	Inhibits FcγRIIA-mediated platelet aggregation, ATP secretion, P-selectin expression and platelet-neutrophil complex formation	Goldmann et al., 2019
		BTK/TEC	GPVI-FcRγ-SYK-LAT-PI3K-BTK/TEC-PLCγ2-PKC	Delays collagen- or CRP-induced platelet aggregation, granule secretion and inside-out activation of αIIbβ3	Bye et al., 2017; [Bibr B150][Bibr B34]; [Bibr B233][Bibr B49]
				Inhibits plaque-induced platelet aggregation in blood under static condition	Busygina et al., 2018
				Prevents platelet thrombus formation in arterially flowing blood on human atherosclerotic plaque homogenates and plaque tissue sections	Busygina et al., 2018
	Ibrutinib IC_50_ 0.35 μM		Unclear	Induces shedding of GPIb-IX complex and αIIbβ3 from the platelet surface	Dobie et al., 2019
				Increases membrane fluidity and ROS production of platelets	Popov et al., 2020
		BTK	αIIbβ3-SYK-PI3K-BTK-PLCγ2	Inhibits platelet spreading on fibrinogen, cytoskeletal assembly and platelet aggregation	Dobie et al., 2019; [Bibr B233][Bibr B49]
			GPIb-IX-SYK-LAT-PI3K-BTK-PLCγ2	Inhibits ristocetin- or VWF-induced platelet adhesion and aggregation	Denzinger et al., 2019; Dobie et al., 2019
			CLEC-2-SYK-LAT-BTK-PLCγ2-NFAT	Inhibits rhodocytin-induced platelet activation and granule secretion	Manne et al., 2015; Dobie et al., 2019
					Nicolson et al., 2021
				Reduces the prevalence of CLEC-2-dependent deep vein thrombosis in a mouse model of inferior vena cava stenosis	Nicolson et al., 2021
			FcγRIIA-BTK-PLCγ2	Inhibits FcγRIIA-mediated platelet aggregation, ATP secretion, P-selectin expression and platelet-neutrophil complex formation	Goldmann et al., 2019
				Prevents *in vivo* platelet aggregation stimulated by CD32 cross-linking in 3 healthy physicians	Goldmann et al., 2019
		BTK/TEC	GPVI-FcRγ-SYK-LAT-PI3K-BTK/TEC-PLCγ2-PKC	Delays collagen- or CRP-induced platelet aggregation, granule secretion and inside-out activation of αIIbβ3	Bye et al., 2017; [Bibr B150][Bibr B34]; [Bibr B233][Bibr B49]
				Inhibits plaque-induced platelet aggregation in blood under static condition	Busygina et al., 2018
				Prevents platelet thrombus formation in arterially flowing blood on human atherosclerotic plaque homogenates and plaque tissue sections	Busygina et al., 2018
		SRC family	GPVI-FcRγ-SRC family kinases	Inhibits thrombus formation in blood from healthy donors on collagen under arterial shear conditions	Bye et al., 2017; Dobie et al., 2019

Bruton’s tyrosine kinase is expressed in megakaryocytes and platelets, but XLA patients do not show a bleeding phenotype (Busygina et al., 2019; Huang et al., 2021). Bleedings observed in CLL and MCL patients that receive ibrutinib or acalabrutinib therapy are mainly attributable to the drug-induced platelet dysfunctions (Bye et al., 2017; Chen et al., 2018; Nicolson et al., 2018; Denzinger et al., 2019; Dmitrieva et al., 2020; Ninomoto et al., 2020). It has been consistently reported that ibrutinib and acalabrutinib significantly delay glycoprotein VI (GPVI)-mediated platelet aggregation in response to collagen or collagen-related peptide (CRP) and that ibrutinib has much higher potency than acalabrutinib (IC_50_ 0.35 *versus* 1.85 μM) on this (Bye et al., 2017; Chen et al., 2018; Nicolson et al., 2018; Busygina et al., 2019; Denzinger et al., 2019; Ninomoto et al., 2020; Series et al., 2019; Zheng et al., 2021). Platelets of XLA patients only show defects in GPVI-mediated platelet activation in response to low collagen concentrations but not to high collagen concentrations (Quek et al., 1998). High collagen concentrations also activate the other kinase TEC expressed in platelets, which compensates for BTK functional deficiency in platelets of XLA patients (Quek et al., 1998; Chen et al., 2018; Busygina et al., 2019). Ibrutinib and acalabrutinib inhibit both BTK and TEC to delay collagen-induced platelet aggregation, granule secretion, and “inside-out” activation of the platelet surface integrin αIIbβ3 via the GPVI-FcRγ-SYK-LAT-PI3K-BTK/TEC-PLCγ2-PKC-Ca^2+^ signaling pathway (Bye et al., 2017; Chen et al., 2018; Nicolson et al., 2018; Busygina et al., 2019; Denzinger et al., 2019; Zheng et al., 2021). Complementary to the signaling events downstream of GPVI activation, fibrinogen binding to the platelet integrin αIIbβ3 invokes a parallel “outside-in” signaling cascade also involving the SYK-PI3K-BTK axis to mediate platelet spreading on fibrinogen, cytoskeletal assembly and platelet aggregation, which is significantly decreased by ibrutinib and acalabrutinib (Dobie et al., 2019; Zheng et al., 2021). Similarly, ibrutinib and acalabrutinib inhibit ristocetin- or botrocetin/von Willebrand factor (VWF)-induced platelet adhesion and aggregation via the GPIb-IX-SYK-LAT-PI3K-BTK-PLCγ2-Ca^2+^ signaling pathway (Busygina et al., 2019; Denzinger et al., 2019; Dobie et al., 2019). In contrast, ibrutinib and acalabrutinib do not compromise platelet activation and aggregation induced by ADP, TRAP6 or arachidonic acid (Busygina et al., 2019; Goldmann et al., 2019; Ninomoto et al., 2020). Collectively, ibrutinib and acalabrutinib specifically inhibit GPVI-mediated platelet activation and aggregation via BTK/TEC-dependent mechanisms and also affect αIIbβ3- and GPIb-IX-mediated signaling in platelets by inhibiting the SYK-PI3K-BTK axis (Table 5), which contribute to the increased bleeding observed in CLL and MCL patients.

Interestingly, mounting evidence suggests that some of the effects of ibrutinib and acalabrutinib on platelets can be harnessed to treat thrombosis-related cardiovascular diseases. Interaction of plaque-derived collagen with GPVI and interaction of VWF with GPIb are essential for thrombus formation on ruptured or eroded atherosclerotic plaques, termed atherothrombosis (Busygina et al., 2018; Denzinger et al., 2019). Both BTK inhibitors do not impair primary hemostasis but do inhibit GPVI-mediated platelet aggregation induced by collagen under blood flow conditions and in blood exposed to human plaque homogenates (Busygina et al., 2018). Ibrutinib and acalabrutinib are also able to prevent platelet thrombus formation in arterially flowing blood on human atherosclerotic plaque homogenates and plaque tissue sections (Busygina et al., 2018). This plaque-selective platelet inhibition was verified in CLL patients taking ibrutinib and in volunteers after much lower and intermittent dosing of ibrutinib (Busygina et al., 2018; Busygina et al., 2019). However, ibrutinib but not acalabrutinib inhibits thrombus formation in blood from healthy donors on collagen under arterial shear conditions due to off-target inhibition of the SRC family kinases by ibrutinib (Bye et al., 2017; Dobie et al., 2019). In line with this, platelets from ibrutinib- but not zanubrutinib-treated CLL patients also exhibit reduced thrombus formation on collagen under arterial flow conditions (Dobie et al., 2019). Intriguingly, low concentrations of ibrutinib and acalabrutinib effectively block the C-type lectin receptor CLEC-2-mediated platelet activation, which demonstrates a critical role in inflammation-driven venous thrombosis but not in hemostasis (Manne et al., 2015; Dobie et al., 2019; Nicolson et al., 2021). Platelets from ibrutinib- or acalabrutinib-treated CLL patients do not aggregate in response to the CLEC-2 agonist rhodocytin and cannot adhere to the CLEC-2 ligand podoplanin under venous flow conditions *ex vivo* (Nicolson et al., 2021). The inhibitory effects of ibrutinib and acalabrutinib on CLEC-2-mediated platelet activation are primarily mediated through specific inhibition of BTK in the SYK-LAT-BTK-PLCγ2-NF-AT pathway and a BTK-dependent positive feedback signaling involving ADP and thromboxane A2, as CLEC-2-mediated platelet activation and aggregation are also blocked by BTK mutations in platelets of XLA patients (Nicolson et al., 2021). *In vivo* administration of ibrutinib reduces the prevalence of CLEC-2-dependent deep vein thrombosis in a mouse model of inferior vena cava stenosis (Nicolson et al., 2021). Based on these findings, BTK inhibitors have been proposed as novel drugs for treating atherothrombosis and thrombo-inflammatory diseases.

Additional potential application of BTK inhibitors on platelet-related diseases includes heparin-induced thrombocytopenia type II (HIT), in which activation of the platelet FcγRIIA (CD32a) is an early and crucial step of disease pathogenesis (Goldmann et al., 2019). Both ibrutinib and acalabrutinib inhibit FcγRIIA-induced platelet aggregation, ATP secretion, P-selectin expression and formation of platelet-neutrophil complexes in blood from healthy donors in response to stimulation by antibody-mediated CD32a cross-linking or sera from HIT patients *in vitro* and *ex vivo*, but ibrutinib exhibits much higher potency than acalabrutinib (IC_50_: 0.08 *versus* 0.38 μM) (Goldmann et al., 2019). A single dose of ibrutinib also prevents *in vivo* platelet aggregation stimulated by CD32 cross-linking in three healthy physicians (Goldmann et al., 2019). Thus, ibrutinib and acalabrutinib can protect against HIT by inhibiting FcγRIIA-BTK-PLCγ2 signaling in platelets and this new rationale warrants testing in patients with HIT (Goldmann et al., 2019).

Taken together, ibrutinib is generally more potent than acalabrutinib at inhibiting GPVI-, αIIbβ3-, GPIb-IX-, CLEC-2- and FcγRIIA-induced platelet aggregation via blocking the BTK/TEC-PLCγ2 signaling axis in platelets (Table 5). Ibrutinib but not acalabrutinib also inhibits thrombus formation on collagen under arterial shear conditions due to off-target inhibition of the SRC family kinases. Although the effects of ibrutinib and acalabrutinib on platelet function may cause undesired bleeding risks in CLL and MCL patients (especially those treated with ibrutinib), some of these effects expand their potential as anti-platelet drugs even at low doses for treating atherothrombosis, thrombo-inflammatory diseases and HIT.

## Innate Lymphoid Cells

To date, there are no studies in the literature reporting the effects of ibrutinib or acalabrutinib on innate lymphoid cells (ILCs), the most recently discovered immune cell subsets. However, ITK is expressed in ILC subsets at a level similar to that detected in NK cells (Eken et al., 2019). The functional importance of ITK in ILCs is highlighted by the evidence that the frequency of ILC2 and ILC3 populations is decreased in the peripheral blood of an *ITK*-deficient patient and that *Itk*^–/–^ mice exhibit a substantial loss of ILC2 in the intestinal lamina propria (Cho et al., 2019; Eken et al., 2019). In this context, it is likely that ibrutinib treatment may affect ILC2 and ILC3 subsets through its off-target inhibition of ITK, which awaits investigation.

## Summary

The clinical success of ibrutinib and acalabrutinib represents a major breakthrough in the treatment of CLL and MCL, and has also revolutionized the treatment options for other B cell malignancies. Compared to ibrutinib, acalabrutinib has improved target specificity and therefore reduced toxicities. Increasing preclinical and clinical evidence indicates that both ibrutinib and acalabrutinib have multifaceted immunomodulatory effects on various immune cell subsets. The shared effects of ibrutinib and acalabrutinib on these immune cell subsets are primarily mediated through inhibition of BTK-dependent signaling pathways of specific immune receptors. These BTK-dependent receptors include adaptive immune receptors (such as BCR and TCR), innate immune receptors (such as TLRs, TREM-1, Dectin-1, NLRP3 and CLEC-2), cytokine receptors (such as GM-CSFR and RANK), chemokine receptors (such as CXCR4 and CXCR5), Fc receptors (such as FcγRIIIA, FcγRIIA and FcεRI) and adhesion molecules (such as GPVI, αIIbβ3 and GPIb-IX). However, ibrutinib also has distinct effects on T cells, NK cells, myeloid cells and platelets through its off-target inhibition of ITK, TEC and the SRC family kinases. Elucidation of these signaling pathways has provided a much better understanding of the mechanisms of action that contribute to the exceptionally high clinical efficacy as well as the unique toxicity profiles of the two drugs observed in CLL and MCL patients. Furthermore, these findings open up new indications for clinical applications of both drugs in a wide variety of human diseases beyond B cell malignancies.

## Author Contributions

PX and SZ have taken the leading roles in designing and writing this manuscript. SG, JJ, ES, JT, JA, BW, and EV have also made significant contributions to writing this manuscript. All authors contributed to the article and approved the submitted version.

## Conflict of Interest

This study was partially supported by a research grant from Acerta Pharma, LLC.

## Publisher’s Note

All claims expressed in this article are solely those of the authors and do not necessarily represent those of their affiliated organizations, or those of the publisher, the editors and the reviewers. Any product that may be evaluated in this article, or claim that may be made by its manufacturer, is not guaranteed or endorsed by the publisher.

## Footnotes

1
www.clinicaltrials.gov
